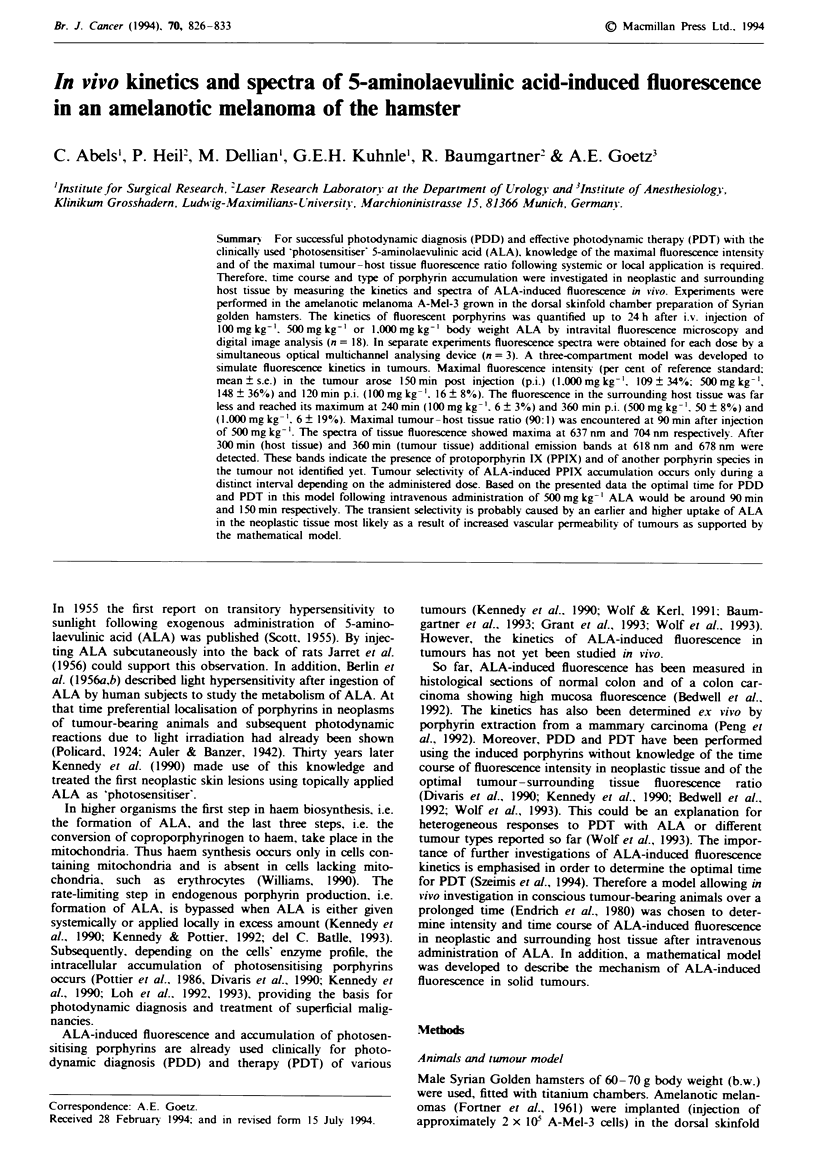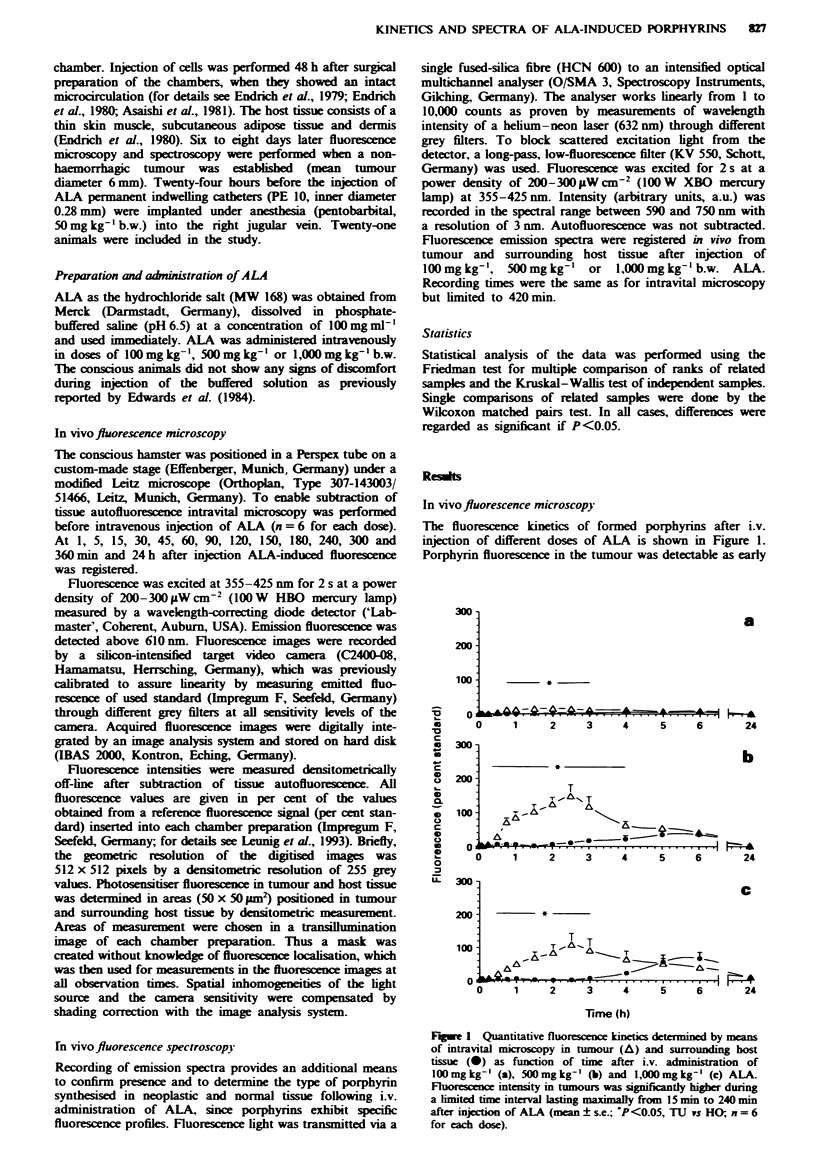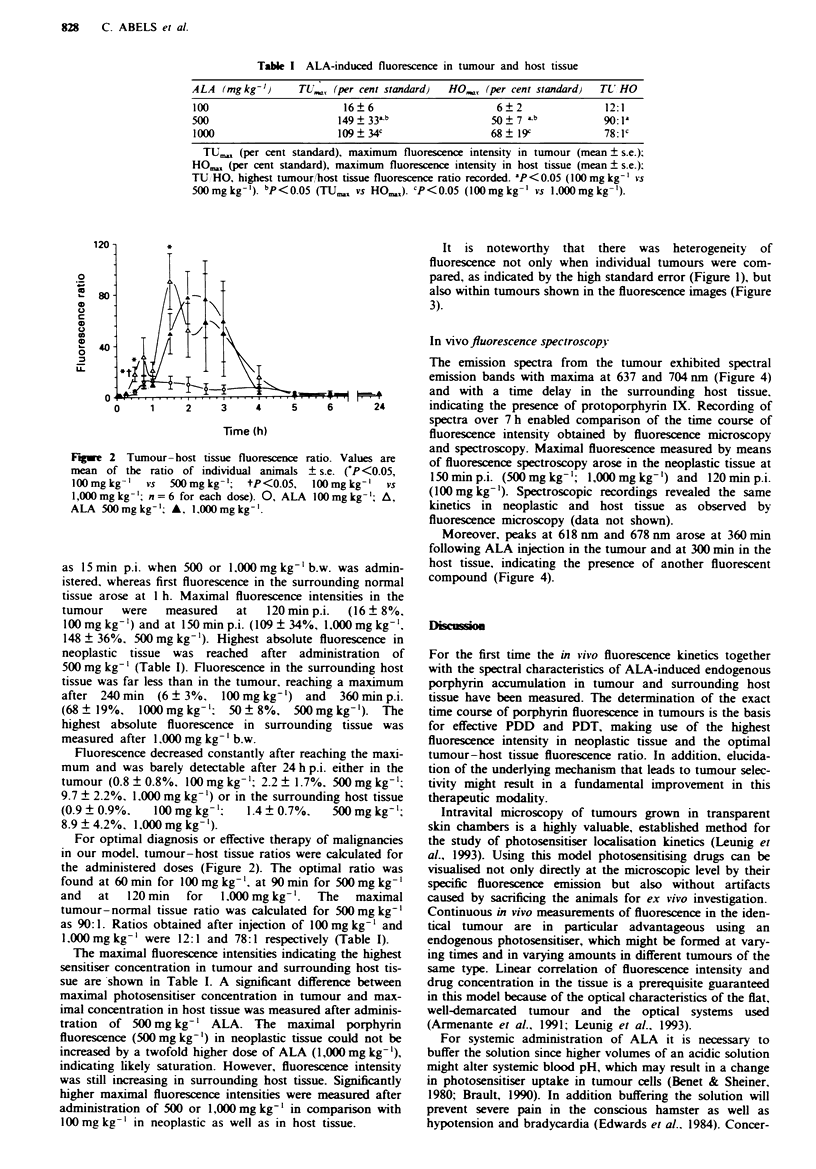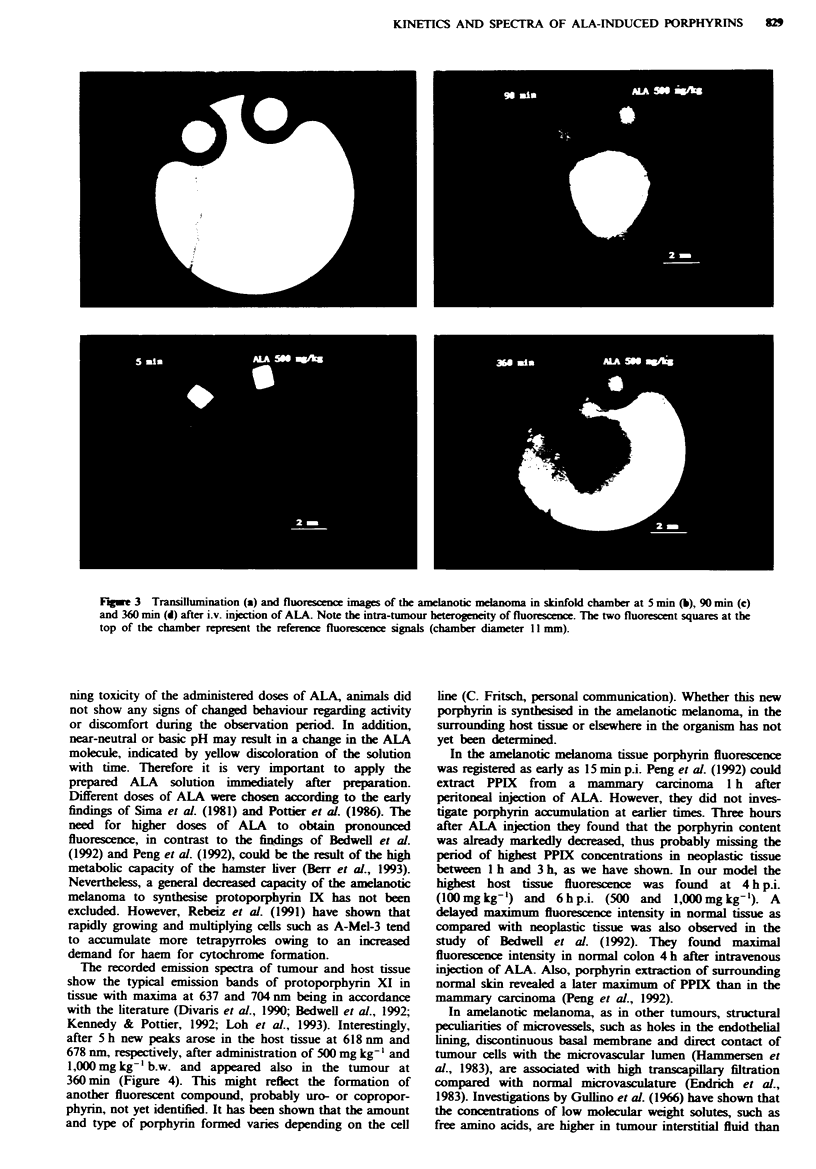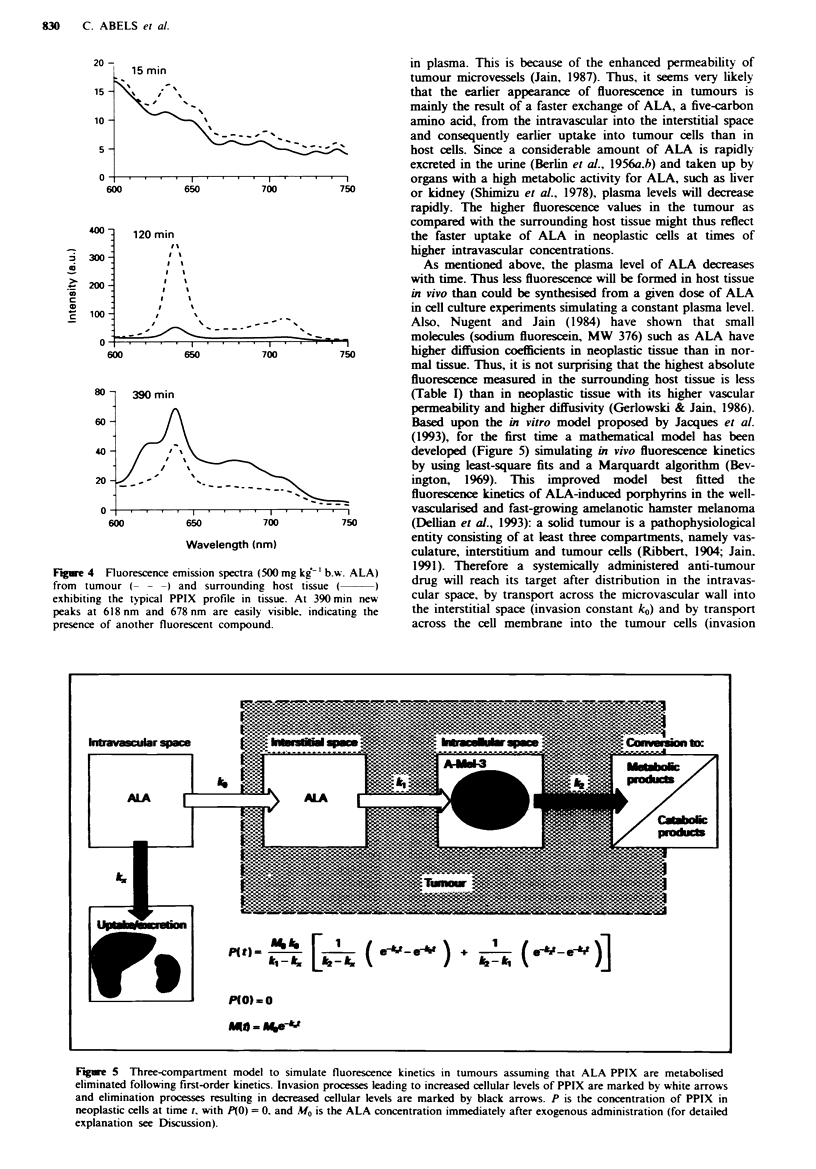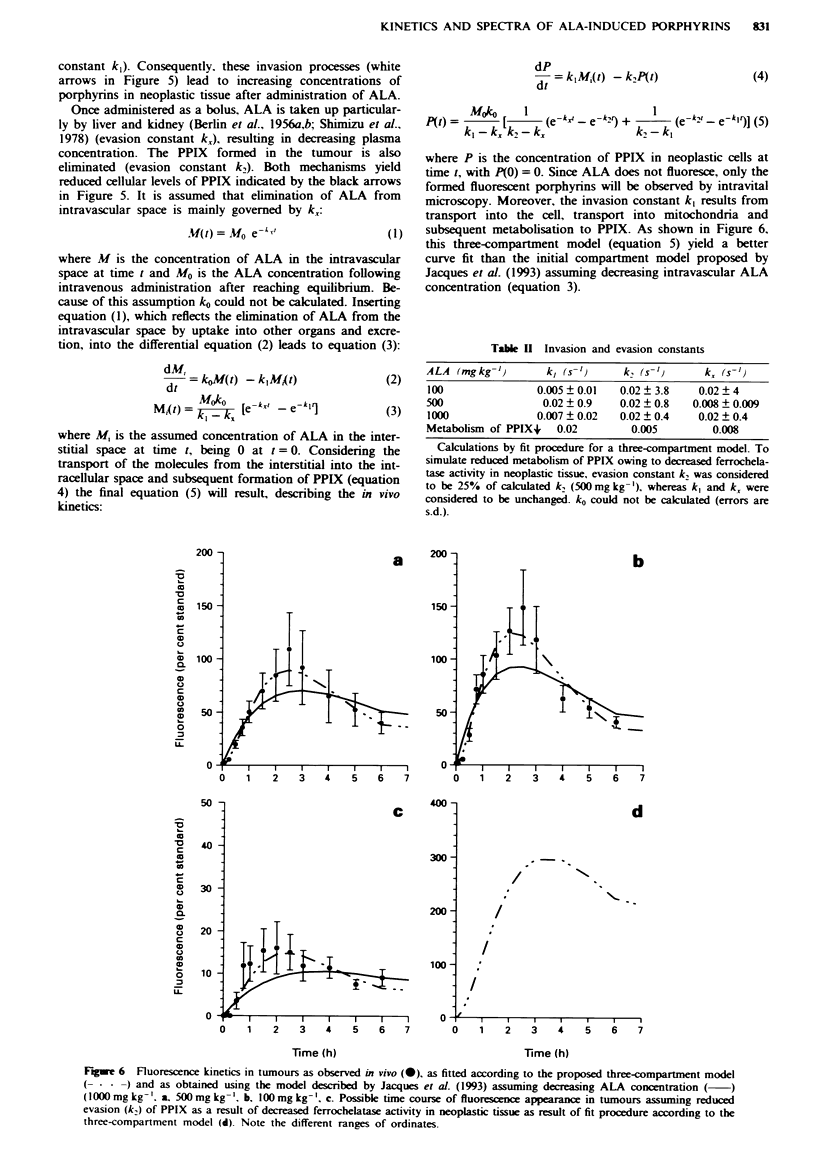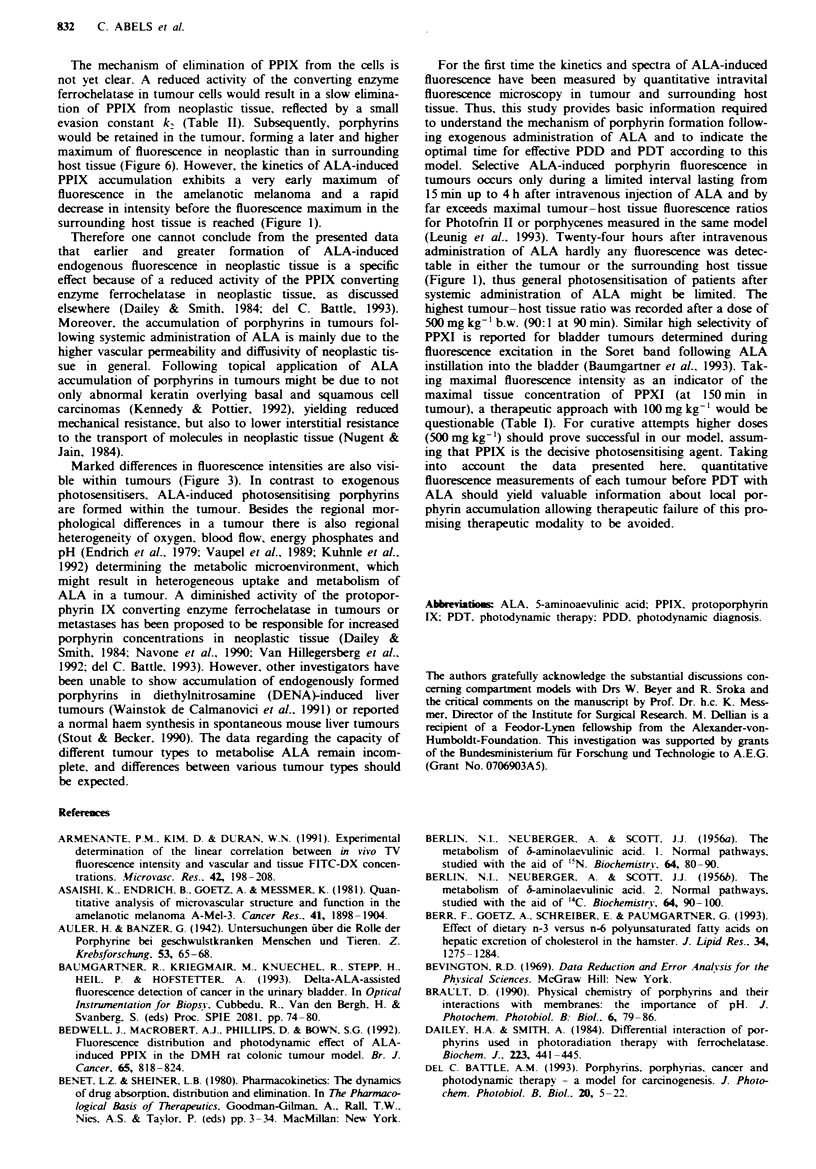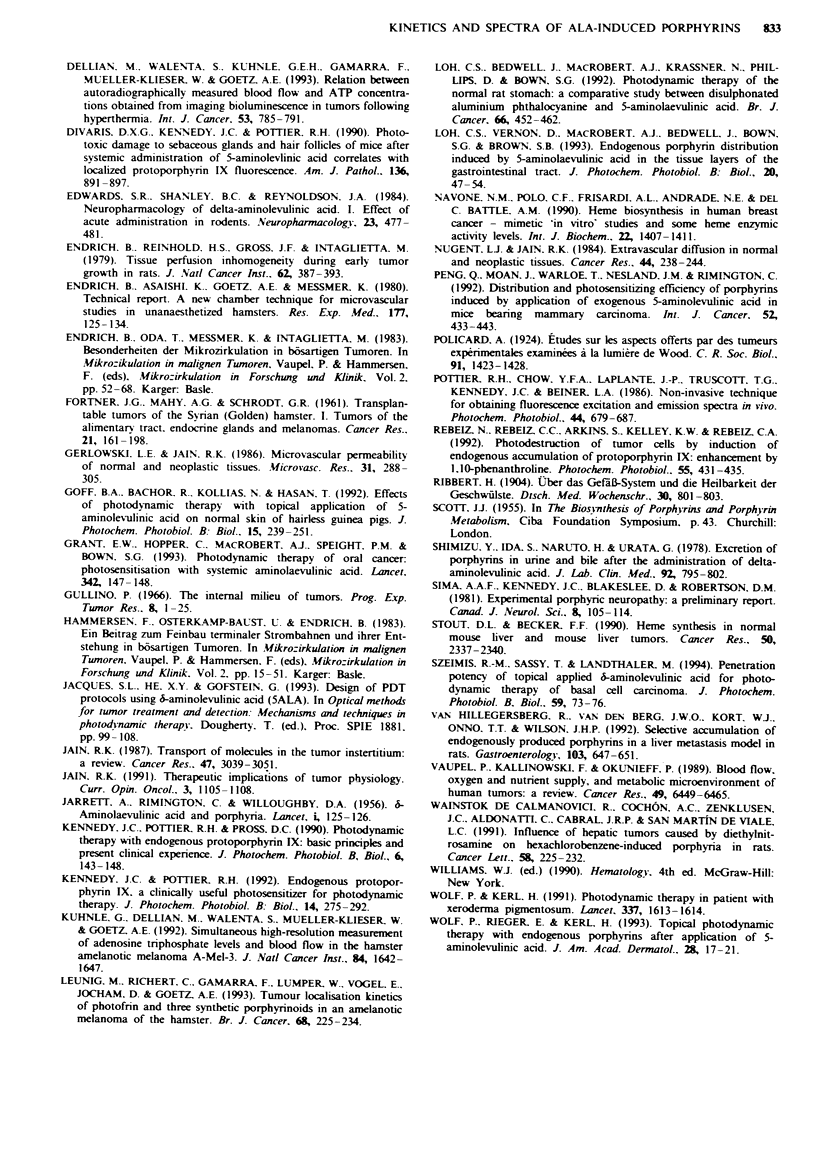# In vivo kinetics and spectra of 5-aminolaevulinic acid-induced fluorescence in an amelanotic melanoma of the hamster.

**DOI:** 10.1038/bjc.1994.406

**Published:** 1994-11

**Authors:** C. Abels, P. Heil, M. Dellian, G. E. Kuhnle, R. Baumgartner, A. E. Goetz

**Affiliations:** Institute for Surgical Research, Klinikum Grosshadern, Ludwig-Maximilians-University, Munich, Germany.

## Abstract

**Images:**


Br. J. Cancer (1994), 70, 826-833 © Macmillan Press Ltd., 1994

### *In vivo* kinetics and spectra of 5-aminolaevulinic acid-induced fluorescence in an amelanotic melanoma of the hamster

C. Abels[1], P. Heil[2], M. Dellian[1], G.E.H. Kuhnle[1], R. Baumgartner[2] & A.E. Goetz[3]

[1]*Institute for Surgical Research,* [2]*Laser Research Laboratory at the Department of Urology and* [3]*Institute of Anesthesiology, Klinikum Grosshadern, Ludwig-Maximilians-University, Marchioninistrasse 15, 81366 Munich, Germany.*

**Summary** For successful photodynamic diagnosis (PDD) and effective photodynamic therapy (PDT) with the clinically used 'photosensitiser' 5-aminolaevulinic acid (ALA), knowledge of the maximal fluorescence intensity and of the maximal tumour-host tissue fluorescence ratio following systemic or local application is required. Therefore, time course and type of porphyrin accumulation were investigated in neoplastic and surrounding host tissue by measuring the kinetics and spectra of ALA-induced fluorescence *in vivo*. Experiments were performed in the amelanotic melanoma A-Mel-3 grown in the dorsal skinfold chamber preparation of Syrian golden hamsters. The kinetics of fluorescent porphyrins was quantified up to 24 h after i.v. injection of 100 mg kg$^{-1}$, 500 mg kg$^{-1}$ or 1,000 mg kg$^{-1}$ body weight ALA by intravital fluorescence microscopy and digital image analysis ($n = 18$). In separate experiments fluorescence spectra were obtained for each dose by a simultaneous optical multichannel analysing device ($n = 3$). A three-compartment model was developed to simulate fluorescence kinetics in tumours. Maximal fluorescence intensity (per cent of reference standard; mean ± s.e.) in the tumour arose 150 min post injection (p.i.) (1,000 mg kg$^{-1}$, 109 ± 34%; 500 mg kg$^{-1}$, 148 ± 36%) and 120 min p.i. (100 mg kg$^{-1}$, 16 ± 8%). The fluorescence in the surrounding host tissue was far less and reached its maximum at 240 min (100 mg kg$^{-1}$, 6 ± 3%) and 360 min p.i. (500 mg kg$^{-1}$, 50 ± 8%) and (1,000 mg kg$^{-1}$, 6 ± 19%). Maximal tumour-host tissue ratio (90:1) was encountered at 90 min after injection of 500 mg kg$^{-1}$. The spectra of tissue fluorescence showed maxima at 637 nm and 704 nm respectively. After 300 min (host tissue) and 360 min (tumour tissue) additional emission bands at 618 nm and 678 nm were detected. These bands indicate the presence of protoporphyrin IX (PPIX) and of another porphyrin species in the tumour not identified yet. Tumour selectivity of ALA-induced PPIX accumulation occurs only during a distinct interval depending on the administered dose. Based on the presented data the optimal time for PDD and PDT in this model following intravenous administration of 500 mg kg$^{-1}$ ALA would be around 90 min and 150 min respectively. The transient selectivity is probably caused by an earlier and higher uptake of ALA in the neoplastic tissue most likely as a result of increased vascular permeability of tumours as supported by the mathematical model.

In 1955 the first report on transitory hypersensitivity to sunlight following exogenous administration of 5-aminolaevulinic acid (ALA) was published (Scott, 1955). By injecting ALA subcutaneously into the back of rats Jarret *et al.* (1956) could support this observation. In addition, Berlin *et al.* (1956*a*,*b*) described light hypersensitivity after ingestion of ALA by human subjects to study the metabolism of ALA. At that time preferential localisation of porphyrins in neoplasms of tumour-bearing animals and subsequent photodynamic reactions due to light irradiation had already been shown (Policard, 1924; Auler & Banzer, 1942). Thirty years later Kennedy *et al.* (1990) made use of this knowledge and treated the first neoplastic skin lesions using topically applied ALA as 'photosensitiser'.

In higher organisms the first step in haem biosynthesis, i.e. the formation of ALA, and the last three steps, i.e. the conversion of coproporphyrinogen to haem, take place in the mitochondria. Thus haem synthesis occurs only in cells containing mitochondria and is absent in cells lacking mitochondria, such as erythrocytes (Williams, 1990). The rate-limiting step in endogenous porphyrin production, i.e. formation of ALA, is bypassed when ALA is either given systemically or applied locally in excess amount (Kennedy *et al.*, 1990; Kennedy & Pottier, 1992; del C. Batlle, 1993). Subsequently, depending on the cells' enzyme profile, the intracellular accumulation of photosensitising porphyrins occurs (Pottier *et al.*, 1986, Divaris *et al.*, 1990; Kennedy *et al.*, 1990; Loh *et al.*, 1992, 1993), providing the basis for photodynamic diagnosis and treatment of superficial malignancies.

ALA-induced fluorescence and accumulation of photosensitising porphyrins are already used clinically for photodynamic diagnosis (PDD) and therapy (PDT) of various tumours (Kennedy *et al.*, 1990; Wolf & Kerl, 1991; Baumgartner *et al.*, 1993; Grant *et al.*, 1993; Wolf *et al.*, 1993). However, the kinetics of ALA-induced fluorescence in tumours has not yet been studied *in vivo*.

So far, ALA-induced fluorescence has been measured in histological sections of normal colon and of a colon carcinoma showing high mucosa fluorescence (Bedwell *et al.*, 1992). The kinetics has also been determined *ex vivo* by porphyrin extraction from a mammary carcinoma (Peng *et al.*, 1992). Moreover, PDD and PDT have been performed using the induced porphyrins without knowledge of the time course of fluorescence intensity in neoplastic tissue and of the optimal tumour-surrounding tissue fluorescence ratio (Divaris *et al.*, 1990; Kennedy *et al.*, 1990; Bedwell *et al.*, 1992; Wolf *et al.*, 1993). This could be an explanation for heterogeneous responses to PDT with ALA or different tumour types reported so far (Wolf *et al.*, 1993). The importance of further investigations of ALA-induced fluorescence kinetics is emphasised in order to determine the optimal time for PDT (Szeimis *et al.*, 1994). Therefore a model allowing *in vivo* investigation in conscious tumour-bearing animals over a prolonged time (Endrich *et al.*, 1980) was chosen to determine intensity and time course of ALA-induced fluorescence in neoplastic and surrounding host tissue after intravenous administration of ALA. In addition, a mathematical model was developed to describe the mechanism of ALA-induced fluorescence in solid tumours.

### Methods

### *Animals and tumour model*

Male Syrian Golden hamsters of 60-70 g body weight (b.w.) were used, fitted with titanium chambers. Amelanotic melanomas (Fortner *et al.*, 1961) were implanted (injection of approximately 2 × 10$^5$ A-Mel-3 cells) in the dorsal skinfold

Correspondence: A.E. Goetz.

Received 28 February 1994; and in revised form 15 July 1994.

chamber. Injection of cells was performed 48 h after surgical preparation of the chambers, when they showed an intact microcirculation (for details see Endrich *et al.*, 1979; Endrich *et al.*, 1980; Asaishi *et al.*, 1981). The host tissue consists of a thin skin muscle, subcutaneous adipose tissue and dermis (Endrich *et al.*, 1980). Six to eight days later fluorescence microscopy and spectroscopy were performed when a non-haemorrhagic tumour was established (mean tumour diameter 6 mm). Twenty-four hours before the injection of ALA permanent indwelling catheters (PE 10, inner diameter 0.28 mm) were implanted under anesthesia (pentobarbital, 50 mg $kg^{-1}$ b.w.) into the right jugular vein. Twenty-one animals were included in the study.

### *Preparation and administration of ALA*

ALA as the hydrochloride salt (MW 168) was obtained from Merck (Darmstadt, Germany), dissolved in phosphate-buffered saline (pH 6.5) at a concentration of 100 mg $ml^{-1}$ and used immediately. ALA was administered intravenously in doses of 100 mg $kg^{-1}$, 500 mg $kg^{-1}$ or 1,000 mg $kg^{-1}$ b.w. The conscious animals did not show any signs of discomfort during injection of the buffered solution as previously reported by Edwards *et al.* (1984).

### In vivo *fluorescence microscopy*

The conscious hamster was positioned in a Perspex tube on a custom-made stage (Effenberger, Munich, Germany) under a modified Leitz microscope (Orthoplan, Type 307-143003/51466, Leitz, Munich, Germany). To enable subtraction of tissue autofluorescence intravital microscopy was performed before intravenous injection of ALA ($n = 6$ for each dose). At 1, 5, 15, 30, 45, 60, 90, 120, 150, 180, 240, 300 and 360 min and 24 h after injection ALA-induced fluorescence was registered.

Fluorescence was excited at 355–425 nm for 2 s at a power density of 200–300 $\mu W\,cm^{-2}$ (100 W HBO mercury lamp) measured by a wavelength-correcting diode detector ('Labmaster', Coherent, Auburn, USA). Emission fluorescence was detected above 610 nm. Fluorescence images were recorded by a silicon-intensified target video camera (C2400-08, Hamamatsu, Herrsching, Germany), which was previously calibrated to assure linearity by measuring emitted fluorescence of used standard (Impregum F, Seefeld, Germany) through different grey filters at all sensitivity levels of the camera. Acquired fluorescence images were digitally integrated by an image analysis system and stored on hard disk (IBAS 2000, Kontron, Eching, Germany).

Fluorescence intensities were measured densitometrically off-line after subtraction of tissue autofluorescence. All fluorescence values are given in per cent of the values obtained from a reference fluorescence signal (per cent standard) inserted into each chamber preparation (Impregum F, Seefeld, Germany; for details see Leunig *et al.*, 1993). Briefly, the geometric resolution of the digitised images was 512 × 512 pixels by a densitometric resolution of 255 grey values. Photosensitiser fluorescence in tumour and host tissue was determined in areas (50 × 50 $\mu m^2$) positioned in tumour and surrounding host tissue by densitometric measurement. Areas of measurement were chosen in a transillumination image of each chamber preparation. Thus a mask was created without knowledge of fluorescence localisation, which was then used for measurements in the fluorescence images at all observation times. Spatial inhomogeneities of the light source and the camera sensitivity were compensated by shading correction with the image analysis system.

### In vivo *fluorescence spectroscopy*

Recording of emission spectra provides an additional means to confirm presence and to determine the type of porphyrin synthesised in neoplastic and normal tissue following i.v. administration of ALA, since porphyrins exhibit specific fluorescence profiles. Fluorescence light was transmitted via a single fused-silica fibre (HCN 600) to an intensified optical multichannel analyser (O/SMA 3, Spectroscopy Instruments, Gilching, Germany). The analyser works linearly from 1 to 10,000 counts as proven by measurements of wavelength intensity of a helium–neon laser (632 nm) through different grey filters. To block scattered excitation light from the detector, a long-pass, low-fluorescence filter (KV 550, Schott, Germany) was used. Fluorescence was excited for 2 s at a power density of 200–300 $\mu W\,cm^{-2}$ (100 W XBO mercury lamp) at 355–425 nm. Intensity (arbitrary units, a.u.) was recorded in the spectral range between 590 and 750 nm with a resolution of 3 nm. Autofluorescence was not subtracted. Fluorescence emission spectra were registered *in vivo* from tumour and surrounding host tissue after injection of 100 mg $kg^{-1}$, 500 mg $kg^{-1}$ or 1,000 mg $kg^{-1}$ b.w. ALA. Recording times were the same as for intravital microscopy but limited to 420 min.

### *Statistics*

Statistical analysis of the data was performed using the Friedman test for multiple comparison of ranks of related samples and the Kruskal–Wallis test of independent samples. Single comparisons of related samples were done by the Wilcoxon matched pairs test. In all cases, differences were regarded as significant if $P < 0.05$.

### Results

### In vivo *fluorescence microscopy*

The fluorescence kinetics of formed porphyrins after i.v. injection of different doses of ALA is shown in Figure 1. Porphyrin fluorescence in the tumour was detectable as early

**Figure 1** Quantitative fluorescence kinetics determined by means of intravital microscopy in tumour (Δ) and surrounding host tissue (●) as function of time after i.v. administration of 100 mg $kg^{-1}$ (**a**), 500 mg $kg^{-1}$ (**b**) and 1,000 mg $kg^{-1}$ (**c**) ALA. Fluorescence intensity in tumours was significantly higher during a limited time interval lasting maximally from 15 min to 240 min after injection of ALA (mean ± s.e.; *$P < 0.05$, TU *vs* HO; $n = 6$ for each dose).

**Table I** ALA-induced fluorescence in tumour and host tissue

| ALA (mg kg$^{-1}$) | $TU_{max}$ (per cent standard) | $HO_{max}$ (per cent standard) | TU/HO |
|---|---|---|---|
| 100 | 16 ± 6 | 6 ± 2 | 12:1 |
| 500 | 149 ± 33$^{a,b}$ | 50 ± 7 $^{a,b}$ | 90:1$^{a}$ |
| 1000 | 109 ± 34$^{c}$ | 68 ± 19$^{c}$ | 78:1$^{c}$ |

$TU_{max}$ (per cent standard), maximum fluorescence intensity in tumour (mean ± s.e.); $HO_{max}$ (per cent standard), maximum fluorescence intensity in host tissue (mean ± s.e.); TU/HO, highest tumour/host tissue fluorescence ratio recorded. $^{a}P<0.05$ (100 mg kg$^{-1}$ *vs* 500 mg kg$^{-1}$). $^{b}P<0.05$ ($TU_{max}$ *vs* $HO_{max}$). $^{c}P<0.05$ (100 mg kg$^{-1}$ *vs* 1,000 mg kg$^{-1}$).

**Figure 2** Tumour-host tissue fluorescence ratio. Values are mean of the ratio of individual animals ± s.e. ($^{*}P<0.05$, 100 mg kg$^{-1}$ *vs* 500 mg kg$^{-1}$; $^{\dagger}P<0.05$, 100 mg kg$^{-1}$ *vs* 1,000 mg kg$^{-1}$; $n = 6$ for each dose). O, ALA 100 mg kg$^{-1}$; Δ, ALA 500 mg kg$^{-1}$; ▲, 1,000 mg kg$^{-1}$.

as 15 min p.i. when 500 or 1,000 mg kg$^{-1}$ b.w. was administered, whereas first fluorescence in the surrounding normal tissue arose at 1 h. Maximal fluorescence intensities in the tumour were measured at 120 min p.i. (16 ± 8%, 100 mg kg$^{-1}$) and at 150 min p.i. (109 ± 34%, 1,000 mg kg$^{-1}$, 148 ± 36%, 500 mg kg$^{-1}$). Highest absolute fluorescence in neoplastic tissue was reached after administration of 500 mg kg$^{-1}$ (Table I). Fluorescence in the surrounding host tissue was far less than in the tumour, reaching a maximum after 240 min (6 ± 3%, 100 mg kg$^{-1}$) and 360 min p.i. (68 ± 19%, 1000 mg kg$^{-1}$; 50 ± 8%, 500 mg kg$^{-1}$). The highest absolute fluorescence in surrounding tissue was measured after 1,000 mg kg$^{-1}$ b.w.

Fluorescence decreased constantly after reaching the maximum and was barely detectable after 24 h p.i. either in the tumour (0.8 ± 0.8%, 100 mg kg$^{-1}$; 2.2 ± 1.7%, 500 mg kg$^{-1}$; 9.7 ± 2.2%, 1,000 mg kg$^{-1}$) or in the surrounding host tissue (0.9 ± 0.9%, 100 mg kg$^{-1}$; 1.4 ± 0.7%, 500 mg kg$^{-1}$; 8.9 ± 4.2%, 1,000 mg kg$^{-1}$).

For optimal diagnosis or effective therapy of malignancies in our model, tumour-host tissue ratios were calculated for the administered doses (Figure 2). The optimal ratio was found at 60 min for 100 mg kg$^{-1}$, at 90 min for 500 mg kg$^{-1}$ and at 120 min for 1,000 mg kg$^{-1}$. The maximal tumour-normal tissue ratio was calculated for 500 mg kg$^{-1}$ as 90:1. Ratios obtained after injection of 100 mg kg$^{-1}$ and 1,000 mg kg$^{-1}$ were 12:1 and 78:1 respectively (Table I).

The maximal fluorescence intensities indicating the highest sensitiser concentration in tumour and surrounding host tissue are shown in Table I. A significant difference between maximal photosensitiser concentration in tumour and maximal concentration in host tissue was measured after administration of 500 mg kg$^{-1}$ ALA. The maximal porphyrin fluorescence (500 mg kg$^{-1}$) in neoplastic tissue could not be increased by a twofold higher dose of ALA (1,000 mg kg$^{-1}$), indicating likely saturation. However, fluorescence intensity was still increasing in surrounding host tissue. Significantly higher maximal fluorescence intensities were measured after administration of 500 or 1,000 mg kg$^{-1}$ in comparison with 100 mg kg$^{-1}$ in neoplastic as well as in host tissue.

It is noteworthy that there was heterogeneity of fluorescence not only when individual tumours were compared, as indicated by the high standard error (Figure 1), but also within tumours shown in the fluorescence images (Figure 3).

### In vivo *fluorescence spectroscopy*

The emission spectra from the tumour exhibited spectral emission bands with maxima at 637 and 704 nm (Figure 4) and with a time delay in the surrounding host tissue, indicating the presence of protoporphyrin IX. Recording of spectra over 7 h enabled comparison of the time course of fluorescence intensity obtained by fluorescence microscopy and spectroscopy. Maximal fluorescence measured by means of fluorescence spectroscopy arose in the neoplastic tissue at 150 min p.i. (500 mg kg$^{-1}$; 1,000 mg kg$^{-1}$) and 120 min p.i. (100 mg kg$^{-1}$). Spectroscopic recordings revealed the same kinetics in neoplastic and host tissue as observed by fluorescence microscopy (data not shown).

Moreover, peaks at 618 nm and 678 nm arose at 360 min following ALA injection in the tumour and at 300 min in the host tissue, indicating the presence of another fluorescent compound (Figure 4).

### Discussion

For the first time the *in vivo* fluorescence kinetics together with the spectral characteristics of ALA-induced endogenous porphyrin accumulation in tumour and surrounding host tissue have been measured. The determination of the exact time course of porphyrin fluorescence in tumours is the basis for effective PDD and PDT, making use of the highest fluorescence intensity in neoplastic tissue and the optimal tumour-host tissue fluorescence ratio. In addition, elucidation of the underlying mechanism that leads to tumour selectivity might result in a fundamental improvement in this therapeutic modality.

Intravital microscopy of tumours grown in transparent skin chambers is a highly valuable, established method for the study of photosensitiser localisation kinetics (Leunig *et al.*, 1993). Using this model photosensitising drugs can be visualised not only directly at the microscopic level by their specific fluorescence emission but also without artifacts caused by sacrificing the animals for *ex vivo* investigation. Continuous *in vivo* measurements of fluorescence in the identical tumour are in particular advantageous using an endogenous photosensitiser, which might be formed at varying times and in varying amounts in different tumours of the same type. Linear correlation of fluorescence intensity and drug concentration in the tissue is a prerequisite guaranteed in this model because of the optical characteristics of the flat, well-demarcated tumour and the optical systems used (Armenante *et al.*, 1991; Leunig *et al.*, 1993).

For systemic administration of ALA it is necessary to buffer the solution since higher volumes of an acidic solution might alter systemic blood pH, which may result in a change in photosensitiser uptake in tumour cells (Benet & Sheiner, 1980; Brault, 1990). In addition buffering the solution will prevent severe pain in the conscious hamster as well as hypotension and bradycardia (Edwards *et al.*, 1984). Concer-

**Figure 3** Transillumination (**a**) and fluorescence images of the amelanotic melanoma in skinfold chamber at 5 min (**b**), 90 min (**c**) and 360 min (**d**) after i.v. injection of ALA. Note the intra-tumour heterogeneity of fluorescence. The two fluorescent squares at the top of the chamber represent the reference fluorescence signals (chamber diameter 11 mm).

ning toxicity of the administered doses of ALA, animals did not show any signs of changed behaviour regarding activity or discomfort during the observation period. In addition, near-neutral or basic pH may result in a change in the ALA molecule, indicated by yellow discoloration of the solution with time. Therefore it is very important to apply the prepared ALA solution immediately after preparation. Different doses of ALA were chosen according to the early findings of Sima *et al.* (1981) and Pottier *et al.* (1986). The need for higher doses of ALA to obtain pronounced fluorescence, in contrast to the findings of Bedwell *et al.* (1992) and Peng *et al.* (1992), could be the result of the high metabolic capacity of the hamster liver (Berr *et al.*, 1993). Nevertheless, a general decreased capacity of the amelanotic melanoma to synthesise protoporphyrin IX has not been excluded. However, Rebeiz *et al.* (1991) have shown that rapidly growing and multiplying cells such as A-Mel-3 tend to accumulate more tetrapyrroles owing to an increased demand for haem for cytochrome formation.

The recorded emission spectra of tumour and host tissue show the typical emission bands of protoporphyrin XI in tissue with maxima at 637 and 704 nm being in accordance with the literature (Divaris *et al.*, 1990; Bedwell *et al.*, 1992; Kennedy & Pottier, 1992; Loh *et al.*, 1993). Interestingly, after 5 h new peaks arose in the host tissue at 618 nm and 678 nm, respectively, after administration of 500 mg $kg^{-1}$ and 1,000 mg $kg^{-1}$ b.w. and appeared also in the tumour at 360 min (Figure 4). This might reflect the formation of another fluorescent compound, probably uro- or coproporphyrin, not yet identified. It has been shown that the amount and type of porphyrin formed varies depending on the cell line (C. Fritsch, personal communication). Whether this new porphyrin is synthesised in the amelanotic melanoma, in the surrounding host tissue or elsewhere in the organism has not yet been determined.

In the amelanotic melanoma tissue porphyrin fluorescence was registered as early as 15 min p.i. Peng *et al.* (1992) could extract PPIX from a mammary carcinoma 1 h after peritoneal injection of ALA. However, they did not investigate porphyrin accumulation at earlier times. Three hours after ALA injection they found that the porphyrin content was already markedly decreased, thus probably missing the period of highest PPIX concentrations in neoplastic tissue between 1 h and 3 h, as we have shown. In our model the highest host tissue fluorescence was found at 4 h p.i. (100 mg $kg^{-1}$) and 6 h p.i. (500 and 1,000 mg $kg^{-1}$). A delayed maximum fluorescence intensity in normal tissue as compared with neoplastic tissue was also observed in the study of Bedwell *et al.* (1992). They found maximal fluorescence intensity in normal colon 4 h after intravenous injection of ALA. Also, porphyrin extraction of surrounding normal skin revealed a later maximum of PPIX than in the mammary carcinoma (Peng *et al.*, 1992).

In amelanotic melanoma, as in other tumours, structural peculiarities of microvessels, such as holes in the endothelial lining, discontinuous basal membrane and direct contact of tumour cells with the microvascular lumen (Hammersen *et al.*, 1983), are associated with high transcapillary filtration compared with normal microvasculature (Endrich *et al.*, 1983). Investigations by Gullino *et al.* (1966) have shown that the concentrations of low molecular weight solutes, such as free amino acids, are higher in tumour interstitial fluid than

**Figure 4** Fluorescence emission spectra (500 mg $kg^{-1}$ b.w. ALA) from tumour (- - -) and surrounding host tissue (———) exhibiting the typical PPIX profile in tissue. At 390 min new peaks at 618 nm and 678 nm are easily visible, indicating the presence of another fluorescent compound.

in plasma. This is because of the enhanced permeability of tumour microvessels (Jain, 1987). Thus, it seems very likely that the earlier appearance of fluorescence in tumours is mainly the result of a faster exchange of ALA, a five-carbon amino acid, from the intravascular into the interstitial space and consequently earlier uptake into tumour cells than in host cells. Since a considerable amount of ALA is rapidly excreted in the urine (Berlin *et al.*, 1956*a*,*b*) and taken up by organs with a high metabolic activity for ALA, such as liver or kidney (Shimizu *et al.*, 1978), plasma levels will decrease rapidly. The higher fluorescence values in the tumour as compared with the surrounding host tissue might thus reflect the faster uptake of ALA in neoplastic cells at times of higher intravascular concentrations.

As mentioned above, the plasma level of ALA decreases with time. Thus less fluorescence will be formed in host tissue *in vivo* than could be synthesised from a given dose of ALA in cell culture experiments simulating a constant plasma level. Also, Nugent and Jain (1984) have shown that small molecules (sodium fluorescein, MW 376) such as ALA have higher diffusion coefficients in neoplastic tissue than in normal tissue. Thus, it is not surprising that the highest absolute fluorescence measured in the surrounding host tissue is less (Table I) than in neoplastic tissue with its higher vascular permeability and higher diffusivity (Gerlowski & Jain, 1986). Based upon the *in vitro* model proposed by Jacques *et al.* (1993), for the first time a mathematical model has been developed (Figure 5) simulating *in vivo* fluorescence kinetics by using least-square fits and a Marquardt algorithm (Bevington, 1969). This improved model best fitted the fluorescence kinetics of ALA-induced porphyrins in the well-vascularised and fast-growing amelanotic hamster melanoma (Dellian *et al.*, 1993): a solid tumour is a pathophysiological entity consisting of at least three compartments, namely vasculature, interstitium and tumour cells (Ribbert, 1904; Jain, 1991). Therefore a systemically administered anti-tumour drug will reach its target after distribution in the intravascular space, by transport across the microvascular wall into the interstitial space (invasion constant $k_0$) and by transport across the cell membrane into the tumour cells (invasion

**Figure 5** Three-compartment model to simulate fluorescence kinetics in tumours assuming that ALA PPIX are metabolised eliminated following first-order kinetics. Invasion processes leading to increased cellular levels of PPIX are marked by white arrows and elimination processes resulting in decreased cellular levels are marked by black arrows. $P$ is the concentration of PPIX in neoplastic cells at time $t$, with $P(0) = 0$, and $M_0$ is the ALA concentration immediately after exogenous administration (for detailed explanation see Discussion).

constant $k_1$). Consequently, these invasion processes (white arrows in Figure 5) lead to increasing concentrations of porphyrins in neoplastic tissue after administration of ALA.

Once administered as a bolus, ALA is taken up particularly by liver and kidney (Berlin *et al.*, 1956*a*,*b*; Shimizu *et al.*, 1978) (evasion constant $k_x$), resulting in decreasing plasma concentration. The PPIX formed in the tumour is also eliminated (evasion constant $k_2$). Both mechanisms yield reduced cellular levels of PPIX indicated by the black arrows in Figure 5. It is assumed that elimination of ALA from intravascular space is mainly governed by $k_x$:

$$M(t) = M_0 \, e^{-k_x t} \qquad (1)$$

where $M$ is the concentration of ALA in the intravascular space at time $t$ and $M_0$ is the ALA concentration following intravenous administration after reaching equilibrium. Because of this assumption $k_0$ could not be calculated. Inserting equation (1), which reflects the elimination of ALA from the intravascular space by uptake into other organs and excretion, into the differential equation (2) leads to equation (3):

$$\frac{dM_i}{dt} = k_0 M(t) - k_1 M_i(t) \qquad (2)$$

$$M_i(t) = \frac{M_0 k_0}{k_1 - k_x} \left[e^{-k_x t} - e^{-k_1 t}\right] \qquad (3)$$

where $M_i$ is the assumed concentration of ALA in the interstitial space at time $t$, being 0 at $t = 0$. Considering the transport of the molecules from the interstitial into the intracellular space and subsequent formation of PPIX (equation 4) the final equation (5) will result, describing the *in vivo* kinetics:

$$\frac{dP}{dt} = k_1 M_i(t) - k_2 P(t) \qquad (4)$$

$$P(t) = \frac{M_0 k_0}{k_1 - k_x} \left[\frac{1}{k_2 - k_x}\left(e^{-k_x t} - e^{-k_2 t}\right) + \frac{1}{k_2 - k_1}\left(e^{-k_2 t} - e^{-k_1 t}\right)\right] \qquad (5)$$

where $P$ is the concentration of PPIX in neoplastic cells at time $t$, with $P(0) = 0$. Since ALA does not fluoresce, only the formed fluorescent porphyrins will be observed by intravital microscopy. Moreover, the invasion constant $k_1$ results from transport into the cell, transport into mitochondria and subsequent metabolisation to PPIX. As shown in Figure 6, this three-compartment model (equation 5) yield a better curve fit than the initial compartment model proposed by Jacques *et al.* (1993) assuming decreasing intravascular ALA concentration (equation 3).

**Table II** Invasion and evasion constants

| *ALA (mg kg⁻¹)* | $k_1$ ($s^{-1}$) | $k_2$ ($s^{-1}$) | $k_x$ ($s^{-1}$) |
|---|---|---|---|
| 100 | 0.005 ± 0.01 | 0.02 ± 3.8 | 0.02 ± 4 |
| 500 | 0.02 ± 0.9 | 0.02 ± 0.8 | 0.008 ± 0.009 |
| 1000 | 0.007 ± 0.02 | 0.02 ± 0.4 | 0.02 ± 0.4 |
| Metabolism of PPIX↓ | 0.02 | 0.005 | 0.008 |

Calculations by fit procedure for a three-compartment model. To simulate reduced metabolism of PPIX owing to decreased ferrochelatase activity in neoplastic tissue, evasion constant $k_2$ was considered to be 25% of calculated $k_2$ (500 mg kg$^{-1}$), whereas $k_1$ and $k_x$ were considered to be unchanged. $k_0$ could not be calculated (errors are s.d.).

**Figure 6** Fluorescence kinetics in tumours as observed *in vivo* (●), as fitted according to the proposed three-compartment model (- · · -) and as obtained using the model described by Jacques *et al.* (1993) assuming decreasing ALA concentration (——) (1000 mg kg$^{-1}$, **a**, 500 mg kg$^{-1}$, **b**, 100 mg kg$^{-1}$, **c**. Possible time course of fluorescence appearance in tumours assuming reduced evasion ($k_2$) of PPIX as a result of decreased ferrochelatase activity in neoplastic tissue as result of fit procedure according to the three-compartment model (**d**). Note the different ranges of ordinates.

The mechanism of elimination of PPIX from the cells is not yet clear. A reduced activity of the converting enzyme ferrochelatase in tumour cells would result in a slow elimination of PPIX from neoplastic tissue, reflected by a small evasion constant $k_2$ (Table II). Subsequently, porphyrins would be retained in the tumour, forming a later and higher maximum of fluorescence in neoplastic than in surrounding host tissue (Figure 6). However, the kinetics of ALA-induced PPIX accumulation exhibits a very early maximum of fluorescence in the amelanotic melanoma and a rapid decrease in intensity before the fluorescence maximum in the surrounding host tissue is reached (Figure 1).

Therefore one cannot conclude from the presented data that earlier and greater formation of ALA-induced endogenous fluorescence in neoplastic tissue is a specific effect because of a reduced activity of the PPIX converting enzyme ferrochelatase in neoplastic tissue, as discussed elsewhere (Dailey & Smith, 1984; del C. Battle, 1993). Moreover, the accumulation of porphyrins in tumours following systemic administration of ALA is mainly due to the higher vascular permeability and diffusivity of neoplastic tissue in general. Following topical application of ALA accumulation of porphyrins in tumours might be due to not only abnormal keratin overlying basal and squamous cell carcinomas (Kennedy & Pottier, 1992), yielding reduced mechanical resistance, but also to lower interstitial resistance to the transport of molecules in neoplastic tissue (Nugent & Jain, 1984).

Marked differences in fluorescence intensities are also visible within tumours (Figure 3). In contrast to exogenous photosensitisers, ALA-induced photosensitising porphyrins are formed within the tumour. Besides the regional morphological differences in a tumour there is also regional heterogeneity of oxygen, blood flow, energy phosphates and pH (Endrich et al., 1979; Vaupel et al., 1989; Kuhnle et al., 1992) determining the metabolic microenvironment, which might result in heterogeneous uptake and metabolism of ALA in a tumour. A diminished activity of the protoporphyrin IX converting enzyme ferrochelatase in tumours or metastases has been proposed to be responsible for increased porphyrin concentrations in neoplastic tissue (Dailey & Smith, 1984; Navone et al., 1990; Van Hillegersberg et al., 1992; del C. Battle, 1993). However, other investigators have been unable to show accumulation of endogenously formed porphyrins in diethylnitrosamine (DENA)-induced liver tumours (Wainstok de Calmanovici et al., 1991) or reported a normal haem synthesis in spontaneous mouse liver tumours (Stout & Becker, 1990). The data regarding the capacity of different tumour types to metabolise ALA remain incomplete, and differences between various tumour types should be expected.

For the first time the kinetics and spectra of ALA-induced fluorescence have been measured by quantitative intravital fluorescence microscopy in tumour and surrounding host tissue. Thus, this study provides basic information required to understand the mechanism of porphyrin formation following exogenous administration of ALA and to indicate the optimal time for effective PDD and PDT according to this model. Selective ALA-induced porphyrin fluorescence in tumours occurs only during a limited interval lasting from 15 min up to 4 h after intravenous injection of ALA and by far exceeds maximal tumour–host tissue fluorescence ratios for Photofrin II or porphycenes measured in the same model (Leunig et al., 1993). Twenty-four hours after intravenous administration of ALA hardly any fluorescence was detectable in either the tumour or the surrounding host tissue (Figure 1), thus general photosensitisation of patients after systemic administration of ALA might be limited. The highest tumour–host tissue ratio was recorded after a dose of 500 mg $kg^{-1}$ b.w. (90:1 at 90 min). Similar high selectivity of PPXI is reported for bladder tumours determined during fluorescence excitation in the Soret band following ALA instillation into the bladder (Baumgartner et al., 1993). Taking maximal fluorescence intensity as an indicator of the maximal tissue concentration of PPXI (at 150 min in tumour), a therapeutic approach with 100 mg $kg^{-1}$ would be questionable (Table I). For curative attempts higher doses (500 mg $kg^{-1}$) should prove successful in our model, assuming that PPIX is the decisive photosensitising agent. Taking into account the data presented here, quantitative fluorescence measurements of each tumour before PDT with ALA should yield valuable information about local porphyrin accumulation allowing therapeutic failure of this promising therapeutic modality to be avoided.

**Abbreviations:** ALA, 5-aminoaevulinic acid; PPIX, protoporphyrin IX; PDT, photodynamic therapy; PDD, photodynamic diagnosis.

The authors gratefully acknowledge the substantial discussions concerning compartment models with Drs W. Beyer and R. Sroka and the critical comments on the manuscript by Prof. Dr. h.c. K. Messmer, Director of the Institute for Surgical Research. M. Dellian is a recipient of a Feodor-Lynen fellowship from the Alexander-von-Humboldt-Foundation. This investigation was supported by grants of the Bundesministerium für Forschung und Technologie to A.E.G. (Grant No. 0706903A5).

### References

ARMENANTE, P.M., KIM, D. & DURAN, W.N. (1991). Experimental determination of the linear correlation between *in vivo* TV fluorescence intensity and vascular and tissue FITC-DX concentrations. *Microvasc. Res.*, **42**, 198–208.

ASAISHI, K., ENDRICH, B., GOETZ, A. & MESSMER, K. (1981). Quantitative analysis of microvascular structure and function in the amelanotic melanoma A-Mel-3. *Cancer Res.*, **41**, 1898–1904.

AULER, H. & BANZER, G. (1942). Untersuchungen über die Rolle der Porphyrine bei geschwulstkranken Menschen und Tieren. *Z. Krebsforschung*, **53**, 65–68.

BAUMGARTNER, R., KRIEGMAIR, M., KNUECHEL, R., STEPP, H., HEIL, P. & HOFSTETTER, A. (1993). Delta-ALA-assisted fluorescence detection of cancer in the urinary bladder. In *Optical Instrumentation for Biopsy*, Cubbedu, R., Van den Bergh, H. & Svanberg, S. (eds) Proc. SPIE 2081, pp. 74–80.

BEDWELL, J., MACROBERT, A.J., PHILLIPS, D. & BOWN, S.G. (1992). Fluorescence distribution and photodynamic effect of ALA-induced PPIX in the DMH rat colonic tumour model. *Br. J. Cancer*, **65**, 818–824.

BENET, L.Z. & SHEINER, L.B. (1980). Pharmacokinetics: The dynamics of drug absorption, distribution and elimination. In *The Pharmacological Basis of Therapeutics*, Goodman-Gilman, A., Rall, T.W., Nies, A.S. & Taylor, P. (eds) pp. 3–34. MacMillan: New York.

BERLIN, N.I., NEUBERGER, A. & SCOTT, J.J. (1956*a*). The metabolism of δ-aminolaevulinic acid. 1. Normal pathways, studied with the aid of $^{15}N$. *Biochemistry*, **64**, 80–90.

BERLIN, N.I., NEUBERGER, A. & SCOTT, J.J. (1956*b*). The metabolism of δ-aminolaevulinic acid. 2. Normal pathways, studied with the aid of $^{14}C$. *Biochemistry*, **64**, 90–100.

BERR, F., GOETZ, A., SCHREIBER, E. & PAUMGARTNER, G. (1993). Effect of dietary n-3 versus n-6 polyunsaturated fatty acids on hepatic excretion of cholesterol in the hamster. *J. Lipid Res.*, **34**, 1275–1284.

BEVINGTON, R.D. (1969). *Data Reduction and Error Analysis for the Physical Sciences*. McGraw Hill: New York.

BRAULT, D. (1990). Physical chemistry of porphyrins and their interactions with membranes: the importance of pH. *J. Photochem. Photobiol. B: Biol.*, **6**, 79–86.

DAILEY, H.A. & SMITH, A. (1984). Differential interaction of porphyrins used in photoradiation therapy with ferrochelatase. *Biochem. J.*, **223**, 441–445.

DEL C. BATTLE, A.M. (1993). Porphyrins, porphyrias, cancer and photodynamic therapy – a model for carcinogenesis. *J. Photochem. Photobiol. B, Biol.*, **20**, 5–22.

DELLIAN, M., WALENTA, S., KUHNLE, G.E.H., GAMARRA, F., MUELLER-KLIESER, W. & GOETZ, A.E. (1993). Relation between autoradiographically measured blood flow and ATP concentrations obtained from imaging bioluminescence in tumors following hyperthermia. *Int. J. Cancer*, **53**, 785-791.

DIVARIS, D.X.G., KENNEDY, J.C. & POTTIER, R.H. (1990). Phototoxic damage to sebaceous glands and hair follicles of mice after systemic administration of 5-aminolevlinic acid correlates with localized protoporphyrin IX fluorescence. *Am. J. Pathol.*, **136**, 891-897.

EDWARDS, S.R., SHANLEY, B.C. & REYNOLDSON, J.A. (1984). Neuropharmacology of delta-aminolevulinic acid. I. Effect of acute administration in rodents. *Neuropharmacology*, **23**, 477-481.

ENDRICH, B., REINHOLD, H.S., GROSS, J.F. & INTAGLIETTA, M. (1979). Tissue perfusion inhomogeneity during early tumor growth in rats. *J. Natl Cancer Inst.*, **62**, 387-393.

ENDRICH, B., ASAISHI, K., GOETZ, A.E. & MESSMER, K. (1980). Technical report. A new chamber technique for microvascular studies in unanaesthetized hamsters. *Res. Exp. Med.*, **177**, 125-134.

ENDRICH, B., ODA, T., MESSMER, K. & INTAGLIETTA, M. (1983). Besonderheiten der Mikrozirkulation in bösartigen Tumoren. In *Mikrozikulation in malignen Tumoren*, Vaupel, P. & Hammersen, F. (eds), *Mikrozirkulation in Forschung und Klinik*, Vol. 2, pp. 52-68. Karger: Basle.

FORTNER, J.G., MAHY, A.G. & SCHRODT, G.R. (1961). Transplantable tumors of the Syrian (Golden) hamster. I. Tumors of the alimentary tract, endocrine glands and melanomas. *Cancer Res.*, **21**, 161-198.

GERLOWSKI, L.E. & JAIN, R.K. (1986). Microvascular permeability of normal and neoplastic tissues. *Microvasc. Res.*, **31**, 288-305.

GOFF, B.A., BACHOR, R., KOLLIAS, N. & HASAN, T. (1992). Effects of photodynamic therapy with topical application of 5-aminolevulinic acid on normal skin of hairless guinea pigs. *J. Photochem. Photobiol. B: Biol.*, **15**, 239-251.

GRANT, E.W., HOPPER, C., MACROBERT, A.J., SPEIGHT, P.M. & BOWN, S.G. (1993). Photodynamic therapy of oral cancer: photosensitisation with systemic aminolaevulinic acid. *Lancet*, **342**, 147-148.

GULLINO, P. (1966). The internal milieu of tumors. *Prog. Exp. Tumor Res.*, **8**, 1-25.

HAMMERSEN, F., OSTERKAMP-BAUST, U. & ENDRICH, B. (1983). Ein Beitrag zum Feinbau terminaler Strombahnen und ihrer Entstehung in bösartigen Tumoren. In *Mikrozirkulation in malignen Tumoren*, Vaupel, P. & Hammersen, F. (eds), *Mikrozirkulation in Forschung und Klinik*, Vol. 2, pp. 15-51. Karger: Basle.

JACQUES, S.L., HE, X.Y. & GOFSTEIN, G. (1993). Design of PDT protocols using δ-aminolevulinic acid (5ALA). In *Optical methods for tumor treatment and detection: Mechanisms and techniques in photodynamic therapy*, Dougherty, T. (ed.), Proc. SPIE 1881, pp. 99-108.

JAIN, R.K. (1987). Transport of molecules in the tumor instertitium: a review. *Cancer Res.*, **47**, 3039-3051.

JAIN, R.K. (1991). Therapeutic implications of tumor physiology. *Curr. Opin. Oncol.*, **3**, 1105-1108.

JARRETT, A., RIMINGTON, C. & WILLOUGHBY, D.A. (1956). δ-Aminolaevulinic acid and porphyria. *Lancet*, **i**, 125-126.

KENNEDY, J.C., POTTIER, R.H. & PROSS, D.C. (1990). Photodynamic therapy with endogenous protoporphyrin IX: basic principles and present clinical experience. *J. Photochem. Photobiol. B, Biol.*, **6**, 143-148.

KENNEDY, J.C. & POTTIER, R.H. (1992). Endogenous protoporphyrin IX, a clinically useful photosensitizer for photodynamic therapy. *J. Photochem. Photobiol. B: Biol.*, **14**, 275-292.

KUHNLE, G., DELLIAN, M., WALENTA, S., MUELLER-KLIESER, W. & GOETZ, A.E. (1992). Simultaneous high-resolution measurement of adenosine triphosphate levels and blood flow in the hamster amelanotic melanoma A-Mel-3. *J. Natl Cancer Inst.*, **84**, 1642-1647.

LEUNIG, M., RICHERT, C., GAMARRA, F., LUMPER, W., VOGEL, E., JOCHAM, D. & GOETZ, A.E. (1993). Tumour localisation kinetics of photofrin and three synthetic porphyrinoids in an amelanotic melanoma of the hamster. *Br. J. Cancer*, **68**, 225-234.

LOH, C.S., BEDWELL, J., MACROBERT, A.J., KRASSNER, N., PHILLIPS, D. & BOWN, S.G. (1992). Photodynamic therapy of the normal rat stomach: a comparative study between disulphonated aluminium phthalocyanine and 5-aminolaevulinic acid. *Br. J. Cancer*, **66**, 452-462.

LOH, C.S., VERNON, D., MACROBERT, A.J., BEDWELL, J., BOWN, S.G. & BROWN, S.B. (1993). Endogenous porphyrin distribution induced by 5-aminolaevulinic acid in the tissue layers of the gastrointestinal tract. *J. Photochem. Photobiol. B: Biol.*, **20**, 47-54.

NAVONE, N.M., POLO, C.F., FRISARDI, A.L., ANDRADE, N.E. & DEL C. BATTLE, A.M. (1990). Heme biosynthesis in human breast cancer – mimetic 'in vitro' studies and some heme enzymic activity levels. *Int. J. Biochem.*, **22**, 1407-1411.

NUGENT, L.J. & JAIN, R.K. (1984). Extravascular diffusion in normal and neoplastic tissues. *Cancer Res.*, **44**, 238-244.

PENG, Q., MOAN, J., WARLOE, T., NESLAND, J.M. & RIMINGTON, C. (1992). Distribution and photosensitizing efficiency of porphyrins induced by application of exogenous 5-aminolevulinic acid in mice bearing mammary carcinoma. *Int. J. Cancer*, **52**, 433-443.

POLICARD, A. (1924). Études sur les aspects offerts par des tumeurs expérimentales examinées à la lumière de Wood. *C. R. Soc. Biol.*, **91**, 1423-1428.

POTTIER, R.H., CHOW, Y.F.A., LAPLANTE, J.-P., TRUSCOTT, T.G., KENNEDY, J.C. & BEINER, L.A. (1986). Non-invasive technique for obtaining fluorescence excitation and emission spectra *in vivo*. *Photochem. Photobiol.*, **44**, 679-687.

REBEIZ, N., REBEIZ, C.C., ARKINS, S., KELLEY, K.W. & REBEIZ, C.A. (1992). Photodestruction of tumor cells by induction of endogenous accumulation of protoporphyrin IX: enhancement by 1,10-phenanthroline. *Photochem. Photobiol.*, **55**, 431-435.

RIBBERT, H. (1904). Über das Gefäß-System und die Heilbarkeit der Geschwülste. *Dtsch. Med. Wochenschr.*, **30**, 801-803.

SCOTT, J.J. (1955). In *The Biosynthesis of Porphyrins and Porphyrin Metabolism*, Ciba Foundation Symposium, p. 43. Churchill: London.

SHIMIZU, Y., IDA, S., NARUTO, H. & URATA, G. (1978). Excretion of porphyrins in urine and bile after the administration of delta-aminolevulinic acid. *J. Lab. Clin. Med.*, **92**, 795-802.

SIMA, A.A.F., KENNEDY, J.C., BLAKESLEE, D. & ROBERTSON, D.M. (1981). Experimental porphyric neuropathy: a preliminary report. *Canad. J. Neurol. Sci.*, **8**, 105-114.

STOUT, D.L. & BECKER, F.F. (1990). Heme synthesis in normal mouse liver and mouse liver tumors. *Cancer Res.*, **50**, 2337-2340.

SZEIMIS, R.-M., SASSY, T. & LANDTHALER, M. (1994). Penetration potency of topical applied δ-aminolevulinic acid for photodynamic therapy of basal cell carcinoma. *J. Photochem. Photobiol. B, Biol.*, **59**, 73-76.

VAN HILLEGERSBERG, R., VAN DEN BERG, J.W.O., KORT, W.J., ONNO, T.T. & WILSON, J.H.P. (1992). Selective accumulation of endogenously produced porphyrins in a liver metastasis model in rats. *Gastroenterology*, **103**, 647-651.

VAUPEL, P., KALLINOWSKI, F. & OKUNIEFF, P. (1989). Blood flow, oxygen and nutrient supply, and metabolic microenvironment of human tumors: a review. *Cancer Res.*, **49**, 6449-6465.

WAINSTOK DE CALMANOVICI, R., COCHÓN, A.C., ZENKLUSEN, J.C., ALDONATTI, C., CABRAL, J.R.P. & SAN MARTÍN DE VIALE, L.C. (1991). Influence of hepatic tumors caused by diethylnitrosamine on hexachlorobenzene-induced porphyria in rats. *Cancer Lett.*, **58**, 225-232.

WILLIAMS, W.J. (ed.) (1990). *Hematology*, 4th ed. McGraw-Hill: New York.

WOLF, P. & KERL, H. (1991). Photodynamic therapy in patient with xeroderma pigmentosum. *Lancet*, **337**, 1613-1614.

WOLF, P., RIEGER, E. & KERL, H. (1993). Topical photodynamic therapy with endogenous porphyrins after application of 5-aminolevulinic acid. *J. Am. Acad. Dermatol.*, **28**, 17-21.